# The Nail Flag Sign: Case Report in a Man with Diverticulitis and Review of Dermatology Flag Sign of the Hair, Skin, and Nails

**DOI:** 10.7759/cureus.2929

**Published:** 2018-07-05

**Authors:** Philip R Cohen

**Affiliations:** 1 Dermatologist, San Diego Family Dermatology, San Diego, USA

**Keywords:** actinic, diabetes, diverticulosis, hair, flag, keratosis, leprosy, nail, sign, vitiligo

## Abstract

A sign is a physical feature or microscopic change which can be observed when the patient or their tissue specimen is evaluated. An astute clinician or pathologist may be able to diagnose a patient’s condition by recognizing this unique morphologic feature or pathological change. In dermatology, the flag sign—alternating transverse bands of the skin or its adnexal structures that are analogous to the alternating colors of the stripes of a flag—has been associated with distinctive disorders of the hair, skin, or nails. The hair flag sign is characterized by alternating horizontal bands of hypopigmentation of the hair (in children with kwashiorkor type of protein-calorie malnutrition) or hyperpigmentation of the hair (in a male child who has intravenously received several cycles of high-dose methotrexate). In skin, the flag sign can be observed microscopically in actinic keratosis, and demonstrates by alternating parakeratosis and orthokeratosis of the stratum corneum—corresponding to the type of hyperkeratosis occurring above the interadnexal epidermis or the ostea of the acrosyringia and acrotrichia. The nail flag sign—noted in some individuals who have diabetes mellitus, diverticulitis, leprosy, or vitiligo—presents with alternating white and pink-red horizontal bands beginning at the proximal nail fold and extending distally to the free edge of the nail plate. A man with chronic diverticulitis and the nail flag sign is reported; he also has a history of congenital leukonychia, actinic keratoses, basal cell carcinoma and psoriasis. In addition, the features of hair, skin, and nail flag signs are reviewed. The detection of the dermatology flag sign can prompt a pathologist to consider the diagnosis of actinic keratosis. Alternating horizontal bands of hair color can indicate protein-calorie malnutrition or an effect of chemotherapy in a child. Certain autoimmune, inflammatory, or infectious conditions in individuals present with white and pink-red horizontal bands on their nails.

## Introduction

An astute clinician or pathologist may be able to recognize morphologic features or pathologic changes and use it to diagnose a patient’s condition [1]. The observation of such signs during the dermatologic evaluation of an individual or the microscopic examination of a skin biopsy can help the physician consider an appropriate diagnosis [2-3]. The flag sign (alternating transverse bands of the skin or its adnexal structures that are analogous to the alternating colors of the stripes of a flag), in dermatology, has been associated with distinctive hair, skin, and nail disorders.

The hair flag sign is characterized by alternating horizontal bands of hypopigmentation of the hair or hyperpigmentation of the hair. The skin flag sign, demonstrated by the alternating parakeratosis and orthokeratosis of the stratum corneum—corresponding to the type of hyperkeratosis occurring above the interadnexal epidermis or the ostea of the acrosyringia and acrotrichia—can be observed microscopically in case of an actinic keratosis. The nail flag sign presents with alternating white and pink-red horizontal bands beginning at the proximal nail fold and extending distally to the free edge of the nail plate [4-11].

A man with the nail flag sign is described in this case report. In addition, this report reviews not only cutaneous diseases, but also those of the skin appendages—such as the hair and nails—that have a corresponding nail sign.

## Case presentation

A 75-year-old man presented for his semiannual total body skin evaluation. His past medical history was significant for diverticulitis of 27 years duration. Three months earlier, the treatment of his abdominal pain had necessitated a sigmoid resection—with a concurrent appendectomy—and an end colostomy with mucous fistula.

He also has anxiety (for which he takes alprazolam, as needed) and mitral valve prolapse (for which he takes diltiazem daily). In addition to his recent operation, his prior surgery includes the repair of an anal fistula with a rectal advancement flap.

His history of skin disease includes a basal cell carcinoma on the right side of his neck that was excised five years ago and actinic keratoses on sun-exposed skin that have been treated with cryotherapy. He developed psoriasis as a middle-aged adult; his psoriasis is limited to less than 5% of his body surface area; individual plaques were treated initially with clobetasol propionate 0.05% cream or solution followed by triamcinolone 0.1% cream, each applied twice daily for three to five days. He also had a history of allergic contact dermatitis to bandaid adhesive and congenital idiopathic leukonychia that has been present since early childhood.

Cutaneous examination showed eight keratotic plaques on the sun-exposed areas of his face, arms, and legs; the actinic keratoses were treated with liquid nitrogen cryotherapy. Psoriasis lesions were also present, appearing as small, red scaly plaques on the chest, arms, and legs. There were no nail plate changes of psoriasis.

**Figure 1:**
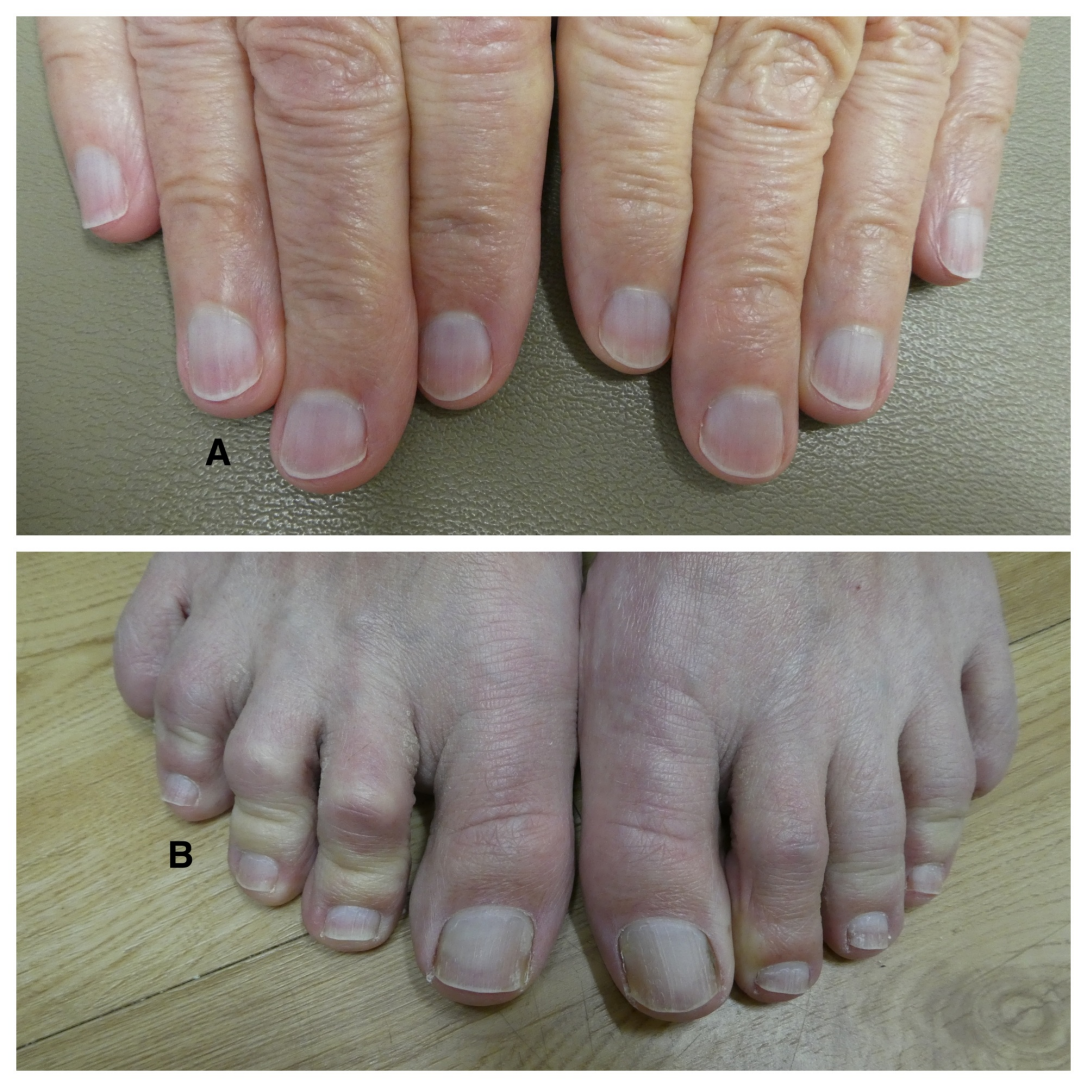
Congenital leukonychia of the fingernails and toenails The fingernails (A) and toenails (B) of a 75-year-old man show diffuse whitening that has been present since childhood consistent with idiopathic leukonychia.

**Figure 2:**
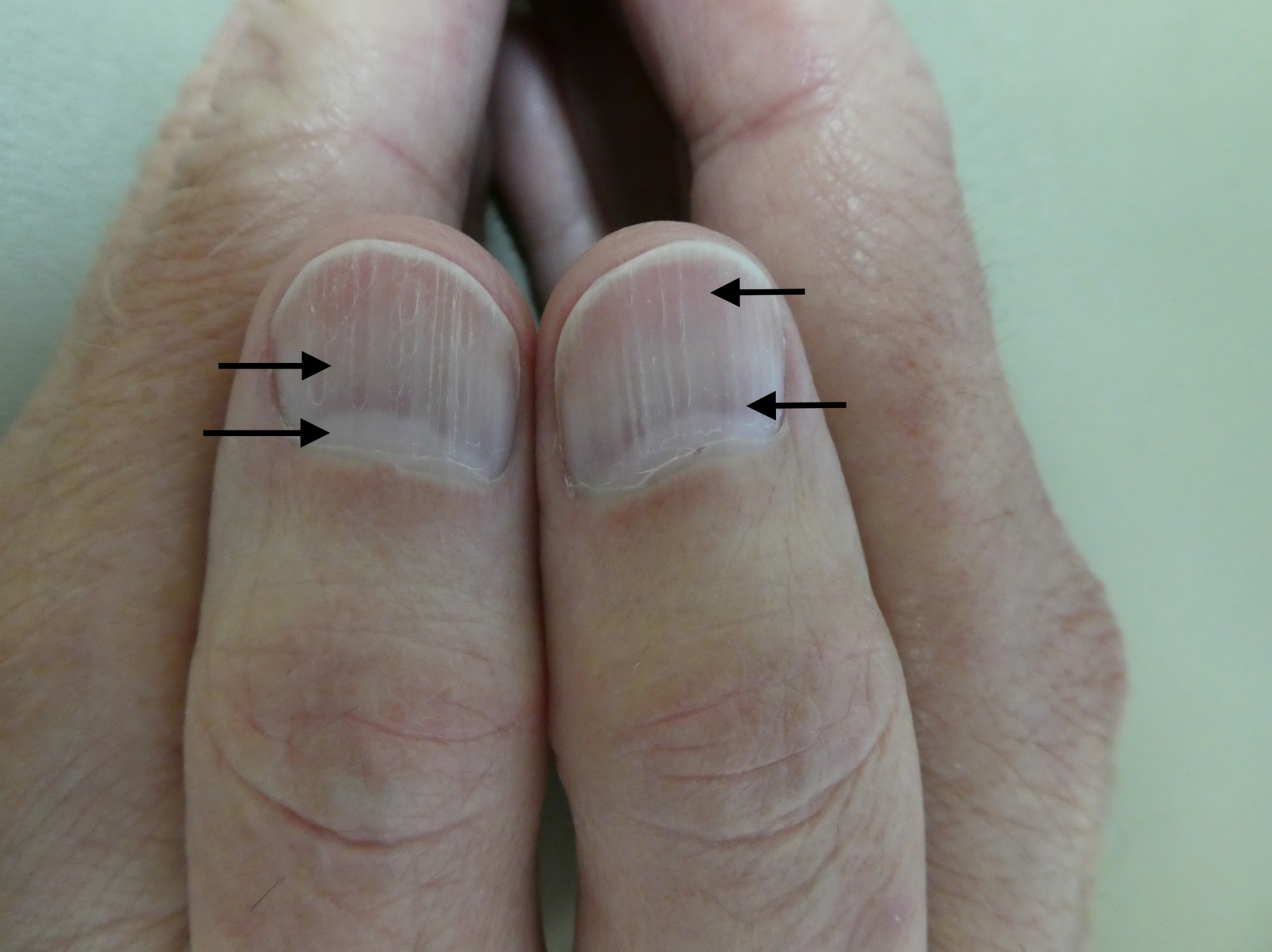
The nail flag sign The thumb nails of a 75-year-old man with a 27-year history of diverticulitis. There are horizontal alternating bands of white (arrows on the left thumb) and red (arrows on the right thumb) beginning at the proximal nail fold and extending to the tip of the nail plate. There are also longitudinal striations on the surface of both nail plates.

Evaluation of his fingernails and toenails confirmed the presence of leukonychia; all of the nail plates on his fingers and toes showed diffuse whitening (Figure 1). His thumbnails also demonstrateed alternating horizontal bands of white and red beginning at the proximal nail fold and extending to the tip of the nail plate: the nail flag sign (Figure 2). In addition, the surfaces of both thumbnail plates had longitudinal striations.

## Discussion

Flags which represent states and nations often have stripes of alternating colors. In some of these flags, such as that of the United States of America, these stripes can take the form of horizontal bands of red and white.

A sign, in medicine, is a physical feature that can be observed when the patient is evaluated by a clinician; similarly, in pathology, it is a unique change that is noted by the pathologist during a microscopic examination of the specimen. Under these circumstances, the sign can either be a component that can be observed under several different conditions or, occasionally, it is a pathognomonic feature of a specific disease. Therefore, the identification of a sign can aid in the consideration or confirmation of a diagnosis.

The flag sign, in the dermatology literature, has been described in three settings: hair, skin, and nails (Table 1). The term was originally coined with regards to scalp hair. Subsequently, the sign was associated as a unique feature of actinic keratoses and, more recently, it was observed on nail plates.

**Table 1:** The flag sign in dermatology

Location	Features	References
Hair	The hypopigmented bands of scalp hair alternating with normal dark hair in children who have the kwashiorkor subtype of protein-calorie malnutrition secondary to chronic infections—such as gastrointestinal or respiratory (tuberculosis)—and infestations—such as roundworm. The hyperpigmented bands of scalp hair alternating with the normal blond hair in a male child corresponding to each intravenous administration of high-dose methotrexate.	[4-8]
Skin	The alternating parakeratosis and orthokeratosis of the hyperkeratotic scale observed microscopically on the stratum corneum of an actinic keratosis and corresponding to the interadnexal epidermis (parakeratosis) and adnexal (acrosyringia and acrotrichia) epidermis (orthokeratosis).	[9]
Nails	The white and pink-red alternating horizontal bands (beginning at the proximal nail fold and extending distally to the free edge of the nail plate) that have been observed in some patients who have either diabetes mellitus, diverticulosis, leprosy or vitiligo.	[10-11]

Protein-calorie malnutrition is most commonly observed in children and presents in two forms: marasmus (a chronic condition usually occurring in the first year of life caused by an insufficiency in both protein and calories) and kwashiorkor (either an acute or chronic condition typically occurring in the second year of life and thereafter caused by a protein-deficient yet calorie-sufficient diet) [4-5]. The flag sign, referred to as “signo de la bandera” by Bradfield and Jelliffe, includes repeated bands of lightened hair color (hypochromotricia) corresponding to distinct periods of malnutrition in children with kwashiorkor [6]. The investigators noted that the kwashiorkor may have been precipitated by not only chronic respiratory (such as tuberculosis) and gastrointestinal infection, but also parasitic (such as round-worm) infestation [4,7].

Subsequently, Wheeland et al. reported the “flag sign of chemotherapy” that they observed in a 4-year-old Caucasian blond-haired boy who developed horizontal hyperpigmented bands of hair that temporally corresponded to the administration of high-dose intravenous methotrexate during the consolidation phase of his treatment for acute lymphoblastic leukemia. These transverse bands of dark pigmentation alternated with the normally blond hair of his scalp, eyebrows, and eyelashes. The researchers postulated that the methotrexate had a direct, albeit temporary, effect on the hair bulb melanocytes; melanosome production and distribution to the medullary and cortical cells of the hair shaft were both increased as a result [8].

An actinic keratosis is a ‘precancerous’ lesion that occurs on sun-exposed sites. It appears as a skin-toned to reddish-brown scaly papule or plaque. Although actinic keratoses are restricted to the epidermis, they have the potential to progress into a non-melanoma skin cancer such as a squamous cell carcinoma [9].

Pathologic changes of an actinic keratosis include atypical keratinocyte with pleomorphic nuclei predominantly occupying the lower portions and the basal layers of the epidermis. Acanthosis, which is an increase in the thickness of the epidermis may also be present. In addition, the upper layers of the epidermis, the stratum cornium, typically demonstrate hyperkeratosis (thickening) consisting not only of parakeratosis (retention of nuclei within the keratinocytes) but also orthokeratosis (epithelial cells without nuclei) [9].

The cutaneous flag sign—characteristic of actinic keratosis—is the pattern of alternating parakeratosis and orthokeratosis that can be observed microscopically. Faulty maturation of the keratinocytes results in parakeratosis atop the interadnexal epidermis. In contrast, the cornified layer above the ostea of the adnexal structures as they extend through the epidermis (such as the acrosyringia of the sweat duct and the acrotrichia of the hair follicle infundibula) shows orthokeratosis [9]. In addition to actinic keratoses, on rare instances, the cutaneous flag sign can be seen as a microscopic feature in other conditions such as irritated human papillomavirus-induced verruca.

The nail flag sign (alternating horizontal bands of white and pink-red discoloration beginning at the proximal nail fold and extending distally to the end of the nail plate) was originally described in both leprosy patients and individuals with diabetes mellitus [10]. Subsequently, it was reported in patients with vitiligo [11]. This report includes the observation of this nail dyschromia in a man with diverticulitis.

El Darouti et al., comparing the changes in the nails of individuals with either leprosy or diabetes, noted a unique pattern of dyschromia—which they termed the ‘flag sign’—on either the fingernails (19 patients) or toenails (five patients) of the 115 patients who had leprosy. The investigators considered the flag sign to be characteristic, albeit not diagnostic, of leprosy. In addition, the researchers speculated that the nail findings resulted from peripheral vascular changes since they observed this feature more commonly in individuals with multibacillary disease (23%; 17 of 81 patients) than those with paucibacillary disease (14%; 5 of 34 patients) [10].

The same researchers also noted the presence of the flag sign of the nails in diabetic patients. They observed alternating white and pink transverse bands on the fingernails in 5% (3 of 60) of the individuals with diabetes mellitus. The other nail changes that were more frequently observed in these patients included longitudinal ridging (42%), dystrophy (32%), and increased curvature (13%) [10].

The nail flag sign was also present in 5 of 91 (6%) vitiligo patients whose nails were evaluated by Anabar et al. In contrast, the dyschromia was not observed in any of the 91 normal healthy age-matched and sex-matched individuals of the control group that was also studied. Other, more common, nail changes occurring in the vitiligo patients included longitudinal striations (44%, 40 of 91 individuals) and absent lunula (17%, 15 of 91 individuals) [11].

The man in this report had a long history of diverticulitis. He mentioned that his nails had always been white since childhood. However, although he was not able to recall when his nails developed the flag sign, he was certain that they did not always have red horizontal bands. Hence, whether his nail dyschromia was associated with his diverticulitis or idiopathic cannot be determined with certainty.

## Conclusions

The flag sign in dermatology refers to distinctive changes in the hair, skin, or nails. The alternating transverse bands on the skin or its adnexal structures are analogous to the alternating colors of the stripes of a flag. The alternating horizontal bands of hypochromotrichia (in kwashiorkor) or darkening pigmentation of the hair (in a patient who had received a high-dose of methotrexate) characterizes the hair flag sign. Similarly, the alternating parakeratosis and orthokeratosis—corresponding to the stratum corneum of the interadnexal epidermis and that overlying the ostea of the acrosyringia and acrotrichia, respectively—comprise the flag sign of an actinic keratosis. And, the nail flag sign—characterized by alternating white and pink-red horizontal bands on the nail plates—has been noted in some individuals who have diabetes mellitus, diverticulitis, leprosy, or vitiligo. Detection of the dermatology flag sign can prompt the pathologist to consider the diagnosis of an actinic keratosis; alternating horizontal bands of hair color in a child could indicate either a protein-calorie malnutrition or recent chemotherapy treatment in a child; and white and pink-red horizontal bands on their nails can indicate certain autoimmune, inflammatory, or infectious conditions in some individuals.
